# Bovine epizootic encephalomyelitis caused by Akabane virus in southern Japan

**DOI:** 10.1186/1746-6148-4-20

**Published:** 2008-06-13

**Authors:** Ryota Kono, Miki Hirata, Masaya Kaji, Yukitoshi Goto, Shogo Ikeda, Tohru Yanase, Tomoko Kato, Shogo Tanaka, Toshiyuki Tsutsui, Tadao Imada, Makoto Yamakawa

**Affiliations:** 1 Kumamoto Central Livestock Hygiene Service Center, Shimomashiki, Kumamoto 861-3205, Japan; 2 Kagoshima Central Livestock Hygiene Service Center, Hioki, Kagoshima 899-2201, Japan; 3 Kyushu Research Station, National Institute of Animal Health, Chuzan, Kagoshima 891-0105, Japan; 4 Epidemiological Research Team, National Institute of Animal Health, Tsukuba, Ibaraki 305-0856, Japan

## Abstract

**Background:**

Akabane virus is a member of the genus *Orthobunyavirus *in the family *Bunyaviridae*. It is transmitted by hematophagous arthropod vectors such as *Culicoides *biting midges and is widely distributed in temperate to tropical regions of the world. The virus is well known as a teratogenic pathogen which causes abortions, stillbirths, premature births and congenital abnormalities with arthrogryposis-hydranencephaly syndrome in cattle, sheep and goats. On the other hand, it is reported that the virus rarely induces encephalomyelitis in cattle by postnatal infection. A first large-scale epidemic of Akabane viral encephalomyelitis in cattle occurred in the southern part of Japan from summer to autumn in 2006. The aim of this study is to define the epidemiological, pathological and virological properties of the disease.

**Results:**

Nonsuppurative encephalomyelitis was observed in cattle that showed neurological symptoms such as astasia, ataxia, opisthotonus and hypersensitivity in beef and dairy farms by histopathological analysis. Akabane viral antigen and genome were consistently detected from the central nervous system of these animals, and the virus was isolated not only from them but also from the blood samples of clinically healthy calves in the epidemic area. The isolates were classified into genogroup I a containing the Iriki strain, which caused encephalitis of calves almost twenty years ago in Japan. Most of the affected cattle possessed the neutralizing antibody against Akabane virus. Seroconversion of the cohabitated and sentinel cattle in the epidemic area was also confirmed during an outbreak of the disease.

**Conclusion:**

The ecological and epidemiological data we have obtained so far demonstrated that the Akabane virus is not endemic in Japan. No evidence of Akabane virus circulation was observed in 2005 through nation-wide serological surveillance, suggesting that a new strain belonging to genogroup I a invaded southern Japan from overseas in the summer of 2006 and caused an unprecedented epizootic of encephalomyelitis mainly in susceptible calves. It will be necessary to reconsider the vaccine strategy to control the disease effectually.

## Background

Akabane virus is classified into the genus *Orthobunyavirus *in the family *Bunyaviridae*. It is widely distributed from the tropical to temperate zones of the world and has been associated with hematophagous arthropod vectors such as *Culicoides *biting midges and mosquitoes, and with ruminants [1-4]. Akabane virus causes epizootic and sporadic outbreaks of abortions, stillbirths, premature births and congenital malformations characterized by arthrogryposis-hydranencephaly syndrome when susceptible pregnant cattle, sheep and goats are infected [5-7]. These outbreaks have been observed in Japan, Korea, Taiwan, Australia, Israel and Turkey [1,8,9], and have repeatedly caused serious economic losses in the livestock industry. It is estimated that more than 42000 abnormal calves were born during the largest outbreak in 1972–75 in Japan. Following that outbreak, attenuated and inactivated vaccines have been developed on the basis of the OBE-1 strain isolated from a naturally infected bovine fetus in 1974 to prevent the disease [10,11].

It is apparent that Akabane virus shares many common features with other members of the genus *Orthobunyavirus *[12]. It possesses a lipid envelope and a genome comprising three segments of a single-stranded, negative-sense RNA designated large (L; 6868 nucleotides), medium (M; 4309 nucleotides) and small (S; 858 nucleotides) [13-15]. The L RNA segment encodes the L protein which contains RNA polymerase activity for replication and transcription of the viral genome. The M RNA segment encodes two viral envelope glycoproteins (Gn and Gc), and a nonstructural (NSm) protein in the form of a precursor polypeptide which is processed by post-translational cleavage. The glycoproteins are responsible for viral neutralization, hemagglutination and attachment to the host cell receptors, while the NSm protein seems to be involved in the process of virus assembly and morphogenesis [16]. The S RNA segment encodes the nucleocapsid (N) protein and a smaller nonstructural (NSs) protein in overlapping reading frames. The N protein shares common antigenic determinants with some other species in the genus. The NSs protein may serve as an alpha/beta interferon antagonist and may be active in the regulation of host protein synthesis and apoptosis [17-19].

Since the strain JaGAr 39 (prototype strain of Akabane virus) was first isolated from *Aedes vexans *and *Culex tritaeniorhynchus *mosquitoes in Gunma prefecture in 1959 [20], many strains have been continuously isolated from the *Culicoides *species and cattle in Japan [21-23]. Recent studies have demonstrated that there are considerable variations in virulence, antigenic properties and nucleotide sequences among the field isolates of Akabane virus [15,22,24-27]. In particular, the Iriki strain is recognized as a typical variant of the virus [22]. A disease characterized by nervous symptoms was observed in ten calves ranging in age from 3 days to 16 months in southern Japan from October to November, 1984. A strain, named 'Iriki', was isolated from the cerebellum of a calf with nonsuppurative encephalitis and identified as an antigenic variant of Akabane virus by the cross-neutralization test. Subsequent seroepidemiological investigation and experimental infection of calves have established that this strain is the causative agent of the disease [22]. Although Akabane virus infection in mature cattle usually results in transient viremia without any clinical signs, a highly pathogenic strain might have the potential to cause encephalitis in cattle by postnatal infection. Small-scale sporadic outbreaks of bovine encephalitis caused by Akabane virus have also been reported in the neighboring countries of Taiwan and Korea [2,28].

In 2006, bovine encephalomyelitis associated with Akabane virus has broken out on a large scale in southern Japan. In this article, we describe the clinical, epidemiological and histopathological features of the disease, and the antigenic and genetic characteristics of the strains isolated from the affected and clinically healthy calves in the epidemic area in order to contribute to the understanding of Akabane viral encephalomyelitis.

## Results

### Clinical and epidemiological features

**Figure 1:**
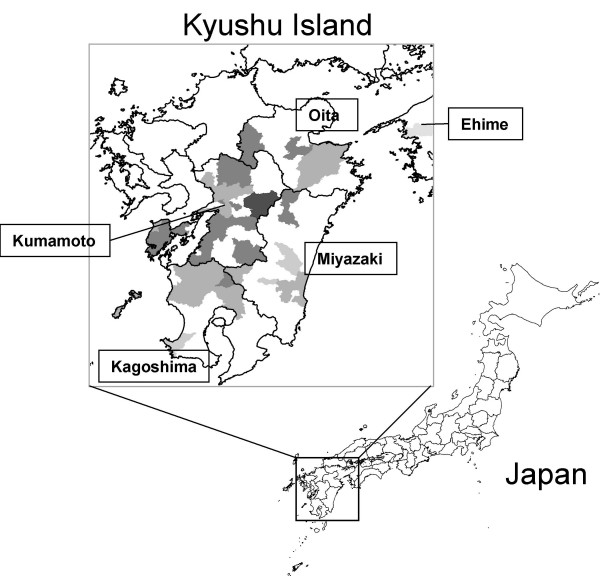
**Districts where bovine encephalomyelitis was observed in 2006**. Dark and light shading corresponds to the month when the first case was observed from August to December 2006. The darkest color shows August and the lightest color shows December.

A total of 180 cattle with neurological disorders were reported in five prefectures (Kumamoto, Kagoshima, Oita, Miyazaki and Ehime) in the southern part of Japan from the end of August to the middle of December in 2006 (Figure 1). Out of 180 affected cattle, 124 (68.9%) and 41 (22.8%) were observed in the Kumamoto and Kagoshima prefectures, respectively, in the Kyushu district. The cattle ranged in age from 4 days to 96 months and showed astasia, dysstasia, ataxia, tremor, nystagmus, opisthotonus and hypersensitivity. Astasia accompanied with paralysis of the hind legs and/or forelegs appeared in most of the affected cattle, while fever and anorexia were rarely detected. These symptoms were found principally in cattle under 24 months of age (140 (77.8%) of 180 cattle). No significant relationship was indicated between the disease and the breed or sex of the cattle.

### Histological findings and immunostaining for Akabane virus antigens in the central nervous system of affected cattle

**Figure 2:**
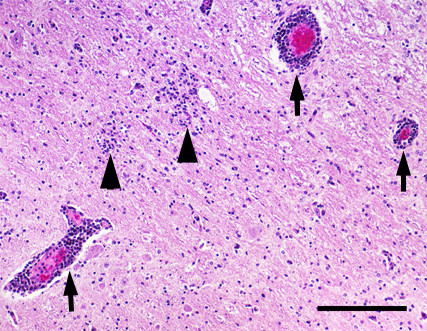
**Perivascular infiltration of mononuclear cells and glial nodules in the formatio reticularis of cerebellopontine**. Arrows indicate the perivascular infiltration of mononuclear cells. Arrow heads point out the glial nodules. Hematoxylin and eosin stain. Bar = 0.5 mm

**Figure 3:**
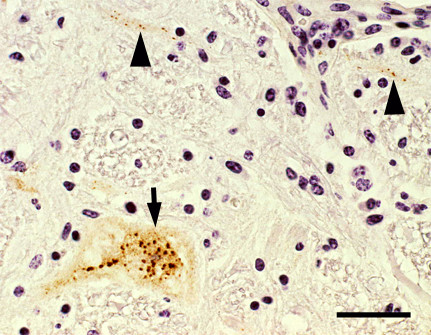
**Detection of Akabane virus antigens in the neuron and nerve axons (Midbrain)**. Akabane virus-positive granules (dark brown color) are observed in the cytoplasm of a neuron (an arrow) and nerve axons (arrow heads). Immunohistochemistry. Bar = 0.1 mm

**Figure 4:**
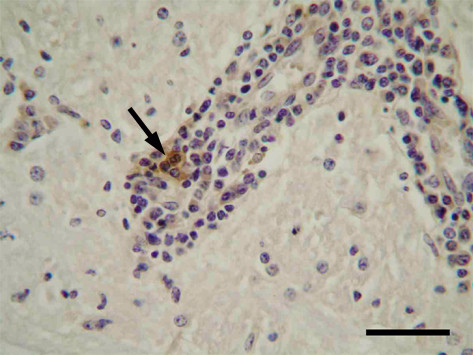
**Detection of Akabane virus antigens in the macrophages aggregated in the perivascular cuffing (Midbrain)**. Akabane virus antigens (dark brown color) are present in the cytoplasm of macrophages (an arrow). Immunohistochemistry. Bar = 0.1 mm

In 72 affected animals found in the Kumamoto and Kagoshima prefectures, no remarkable gross changes were observed in the brain, spinal cord, skeletal muscles or other organs. However, typical nonsuppurative encephalomyelitis characterized by severe to mild perivascular infiltration of mononuclear cells, glial nodules consisting of microglia (Figure 2), and neuronal degeneration and/or necrosis were considerably accumulated in the midbrain, cerebellopontine and medulla oblongata, but mildly in the cerebrum and cerebellum in most of the affected animals. In addition, neuronophagia was occasionally detected in the ventral horns of the spinal cord with moderate inflammatory changes. Strong immunoreactivity for Akabane virus antigens was mainly observed within the cytoplasm of neurons and nerve axons (Figure 3), and occasionally in vascular endothelial cells and macrophages aggregated in the perivascular cuffing (Figure 4) shown in the brain stem area of the affected animals. The intensity and number of focal areas of positive immunoreactivity for the virus antigens tended to increase with the severity of the inflammatory reaction.

### RT-PCR detection and isolation of Akabane virus

Specific PCR products of 389 base pairs were detected in 39 (54.2%) of 72 cattle with nonsuppurative encephalomyelitis. Finally, seven virus strains were isolated from these PCR-positive specimens by HmLu-1 cell cultures. Four strains were also isolated from the blood or plasma of clinically healthy calves collected between September and October on the affected farms. Furthermore, a strain designated KSB-3/P/06 was isolated from the healthy sentinel cattle in Kyushu Research Station, National Institute of Animal Health, in Kagoshima prefecture during an outbreak of the disease. All 12 strains listed in Table 1 were identified as Akabane virus by the immunobinding assay.

**Table 1:** Akabane virus strains isolated during an outbreak of bovine encephalomyelitis and used in this study

			Source			
Strain	Passage level^a^	Geographical origin				
			Specimen for isolation	Date collected	Symptom	Profile of cattle
KM-1/Br/06	Sm1, HL4	Kumamoto	Brain	3 October, 2006	Astasia, Tachypnea	Holstein, 4 days old
KM-2/Br/06	HL4	Kumamoto	Brain	28 September, 2006	Astasia, Tachypnea, Tremor, Opisthotonus	Holstein, female, 4 months old
KM-3/Ce/06	HL3	Kumamoto	Cerebellum	19 October, 2006	Astasia	Holstein, 5 months old
KM-6/Br/06	HL3	Kumamoto	Brain stem	26 October, 2006	Astasia	Holstein, female, 19 months old
KM-7/Pt/06	HL4	Kumamoto	Spinal cord (Pars thoracia)	26 October, 2006	Astasia	Holstein, female, 19 months old
KM-8/Br/06	HL4	Kumamoto	Brain stem	18 October, 2006	Astasia	Holstein, female, 16 months old
KM-9/B/06	HL3	Kumamoto	Blood	30 October, 2006	No symptoms were observed when a blood sample was taken. Astasia appeared on 13 November, 2006.	Holstein, 3 months old
KM-10/B/06	HL3	Kumamoto	Blood	September, 2006	No symptoms	
KM-11/B/06	HL2	Kumamoto	Blood	September, 2006	No symptoms	
KS-1/P/06	HL4	Kagoshima	Plasma	12 October, 2006	No symptoms	Japanese Black, castrated male, 10 months old
KS-2/Mo/06	HL5	Kagoshima	Brain stem (Medulla oblongata)	18 October, 2006	Dysstasia, Ataxia	Japanese Black, castrated male, 6 months old
KSB-3/P/06	BHK4	Kagoshima	Plasma	27 October, 2006	No symptoms	Japanese Black, female, 24 months old

^a ^Letters indicate cell lines and numbers indicate number of passages. HL, HmLu-1 cells; BHK, BHK-21 cells; Sm, suckling-mouse brain (only for isolation of KM-1/Br/06).

### Antigenic characterization of the Akabane virus isolates

**Figure 5:**
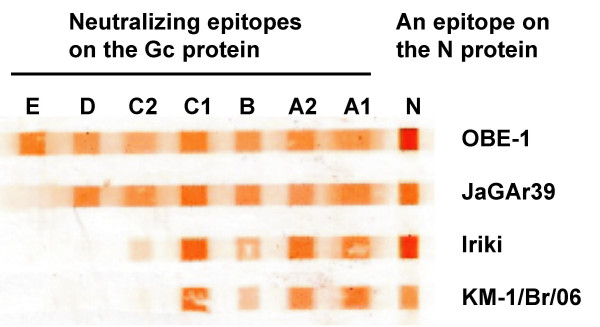
**Reaction of the Akabane virus isolates with monoclonal antibodies in the immunobinding assay**. Akabane virus strains blotted onto the Immobilon-P membrane were reacted with monoclonal antibodies against the OBE-1 strain. The OBE-1, JaGAr39 and Iriki strains in antigenic groups 1, 2 and 3, respectively, reported by Yoshida and Tsuda [25] were used as references.

All of the isolates reacted clearly with a monoclonal antibody to the N protein and four of seven neutralizing monoclonal antibodies recognizing epitopes A1, A2, B and C1 on the Gc protein of the Akabane virus OBE-1 strain [25]. The result of an immunobinding assay for the KM-1/Br/06, a representative strain of 2006 isolates, was shown in Figure 5. The reaction pattern was almost the same as that of the Iriki strain and was classified in antigenic group 3 as described by Yoshida and Tsuda [25].

Further antigenic comparison between one of the isolates, KM-1/Br/06 strain, and the OBE-1 and Iriki strains was performed by the cross-neutralization test using rabbit antisera to these three strains. As demonstrated by the immunobinding assay, the KM-1/Br/06 strain was antigenically identical to the Iriki strain, but was distant from the OBE-1 strain (Table 2). The OBE-1 strain has been neutralized by antibodies against the Iriki and KM-1/Br/06 strains as well as homologous strains.

**Table 2:** Antigenic comparison among Akabane virus strains by cross-neutralization test

Strain	Antibody titer of immune serum		
	
	KM-1/Br/06	Iriki	OBE-1
KM-1/Br/06	2048	2048	64
Iriki	1024	2048	64
OBE-1	2048	2048	512

### Serological surveys

Serological investigations in the Kumamoto and Kagoshima prefectures indicated that 117 (98.3%) of 119 cattle with neurological symptoms and 113 (74.3%) of 152 cohabitated cattle without neurological symptoms in the case farms possessed the neutralizing antibody against Akabane virus. Annual serological surveillance of arthropod-borne viruses demonstrated that 60 (45.5%) of 132 healthy sentinel calves in both prefectures exhibited seroconversion to Akabane virus between September and November in 2006. No seroconversions to the other bovine arthropod-borne viruses, such as Aino, bluetongue, Chuzan, Ibaraki and bovine ephemeral fever viruses, were observed in these sentinel calves this year.

### Sequence comparisons and phylogenetic analyses of the S and M RNA genomic segments

Nucleotide sequences of the S and M RNA segments of isolates were determined, and their open reading frames (ORFs) were compared respectively with those of the Akabane virus strains previously reported [27]. The ORF sequence of the S RNA segment (699 nucleotides) was highly conserved among 12 strains isolated during an outbreak of bovine encephalomyelitis (99.6–100% identity). The sequence identities among one representative strain of 2006 isolates (KM-1/Br/06), Iriki, OBE-1 and the prototype strain JaGAr39 ranged from 95.9 to 99.0% at the nucleotide level and from 99.1 to 100% at the amino acid level (Table 3). The strains KM-1/Br/06, KM-2/Br/06, KS-2/Mo/06 and KSB-3/P/06 were selected for further analysis of the ORF sequence of the M RNA segment (4203 nucleotides) encoding the neutralizing antigen. The nucleotide and amino acid sequences of the M RNA segment were also well conserved among these four strains from 2006 (99.9% and 99.9–100% identity in the nucleotide and amino acid sequences, respectively). As presented in Table 4, the representative strain, KM-1/Br/06, was more closely related to the Iriki strain than to the OBE-1 and JaGAr39 strains.

**Table 3:** Comparison of the S RNA segment (ORF region) among Akabane virus strains

Strain	KM-1/Br/06	Iriki	OBE-1	JaGAr39
Nucleotide sequence identity (%)				
KM-1/Br/06		97.1	95.9	96.9
Iriki	99.6		97.4	98.1
OBE-1	99.1	99.6		99.0
JaGAr39	99.1	99.6	100	
Amino acid sequence identity (%)				

**Table 4:** Comparison of the M RNA segment (ORF region) among Akabane virus strains

Strain	KM-1/Br/06	Iriki	OBE-1	JaGAr39
Nucleotide sequence identity (%)				
KM-1/Br/06		96.1	88.8	89.5
Iriki	98.0		90.2	90.7
OBE-1	94.8	95.4		96.3
JaGAr39	95.3	95.9	98.0	
Amino acid sequence identity (%)				

**Figure 6:**
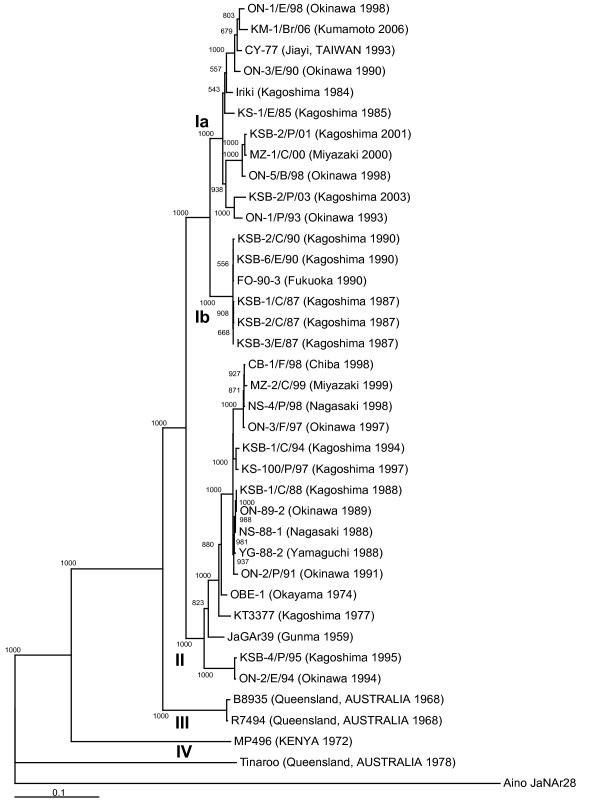
**Phylogenetic profile of the Akabane virus strains based on the sequence of the M RNA segment**. The ORF nucleotide sequences of the M RNA segment (4203 nucleotides) were aligned, and a phylogenetic tree was constructed by the neighbor-joining method. The corresponding nucleotide sequence of Aino virus strain JaNAr28 in the genus *Orthobunyavirus *was used as an outgroup to root the tree. The percentage bootstrap values calculated from 1000 replications are indicated above the internal nodes. The scale represents the 10% nucleotide sequence divergence. The geographical origin and year of isolation of the strains are given in the parentheses.

To define the genetic relationships between the strains isolated in 2006 and the previous isolates of the Akabane virus, the phylogenetic trees of these two segments were constructed. As previously reported [27], the field isolates of Akabane virus were segregated into four distinct lineages (genogroups I – IV) based on the sequence of an M RNA segment. Genogroup I was subdivided into two monophyletic clusters, I a and I b. As shown in Figure 6, the KM-1/Br/06 strain was classified into subgroup I a containing the Iriki strain. The phylogenetic tree of the S RNA segment was almost the same as that of the M RNA segment (data not shown) [26,27].

## Discussion

An epizootic of the disease with neurological disorders in cattle was observed in the southern part of Japan from summer to autumn in 2006. It was initially suggested that the disease was associated with some neurotropic arthropod-borne viruses due to its seasonal occurrence over an extensive area and to the histopathological findings of the affected cattle. We eventually identified Akabane virus as the cause of the disease, as in that observed in 1984 [22], through the detection of the viral antigen and genome in the clinical samples, the isolation of the virus from the affected and clinically healthy cattle in the epidemic area and serological evidence.

Diseases caused by various arthropod-borne viruses, such as Akabane, Aino, Chuzan, Ibaraki and bovine ephemeral fever viruses, have been recognized as major obstacles to the production of beef and dairy cattle in Japan. Above all, Akabane virus is considered to be the most important because the virus circulation has been observed almost every year for over four decades since 1959. Seroconversion of sentinel calves has always been confirmed after the rainy season (June to July) through nation-wide serological surveillance of bovine arthropod-borne viruses. Entomological investigations in southern Japan continuing over two decades indicate that *Culicoides oxystoma *is the principal vector and that bovine arthropod-borne viruses have been isolated between summer and autumn (August to November) when adult midges are abundant [23]. Molecular analyses of the field isolates of Akabane virus collected from 1959 to the present in Japan and phylogenetic comparisons with isolates from Taiwan, Israel, Australia and Kenya demonstrated that the virus circulating widely in Asia seems to have evolved in a common gene pool and can clearly be differentiated from strains existing in Oceania and Africa [26,27]. Given that Akabane virus is unlikely to be persistent in the winter season in Japan, these findings may suggest that the virus is introduced repeatedly from lower latitude areas (tropical and subtropical zones) in Asia, where the climatic conditions are more suitable, by infected *Culicoides *biting midges carried on the seasonal wind over long distances [23,29]. A single strain adaptable to the Japanese environment might spread transiently and cause the disease to recur seasonally [23,26]. However, the possibility of viral overwintering in *Culicoides *larvae by vertical transmission should be confirmed by further investigations of virus-vector interactions.

No evidence of Akabane virus circulation was observed in Japan in 2005 via nation-wide surveillance using sentinel animals. Therefore, it is considered that a new strain initially invaded from overseas into Kumamoto prefecture, where the first clinical case was reported on 30 August in 2006, and then was spread throughout the southern part of Japan by *Culicoides *biting midges until late autumn. The virus finally caused an unprecedented outbreak of encephalomyelitis, mainly in susceptible calves. The absence of the virus circulation in 2005 may have increased the number of susceptible animals without antibody against Akabane virus (data not shown). This may be one of the factors that facilitated a large-scale outbreak of the disease.

There are four Akabane virus genogroups, of which two groups, I and II, are extant in Japan [26,27], whereas the field isolates can be separated into at least five antigenic groups by an immunobinding assay using neutralizing antibodies [25]. Although several discrepancies exist between the genetic and antigenic classifications, the isolates in the antigenic group 3 are consistently included in subgenogroup I a [27]. Twelve strains isolated during 2006 were classified into subgenogroup I a together with the Iriki strain which caused encephalitis in calves in 1984. When introduced by intracerebral inoculation, the Iriki strain produced severe nervous symptoms in calves similar to those observed in natural infection [22]. Interestingly, the histopathological findings in this report were also quite similar to those in cattle infected with the Akabane virus Iriki strain [2,22]. In contrast, the JaGAr 39 and OBE-1 strains belonging to genogroup II caused only slight or no nervous symptoms in the intracerebrally infected calves [30]. Although more experimental data using some other strains and more clinical data in the field will be required to confirm precise relationship between pathogenicity and genogroups, these previous experiments and the epizootic of encephalomyelitis in 2006 suggest that the neurovirulence of strains in genogroup I a is much stronger than that of strains in genogroup II.

The KM-1/Br/06 strain was isolated from a 4-day-old calf with nervous symptoms (Table 1), indicating the vertical transmission of the virus from the infected dam to the fetus. We have therefore traced any abnormal births of cattle in the epidemic area. Consequently, 16 cases of premature births and congenital abnormalities related to Akabane virus have been confirmed from October 2006 through April 2007. Viral genome identical to that of the isolates in 2006 was detected in four calves accompanied by nonsuppurative encephalomyelitis (data not shown). Akabane viral antigen was also found in the brain of one of four PCR-positive calves, but unfortunately no viruses were isolated from these calves. The Akabane virus strains belonging to genogroup I a might be involved not only in encephalomyelitis by postnatal infection but also in conventional teratogenicity by transplacental infection. These strains have been frequently isolated in Japan since 2000 [26,27]. An antigenic difference was recognized between the OBE-1 (the origin of vaccine strain TS-C2) and KM-1/Br/06 strains as shown in this study. It is, therefore, better to develop a novel vaccine for proper prevention and control of the disease. The newly isolated KM-1/Br/06 strain will be one of the candidates for the advanced vaccine. It will also be important to establish surveillance system in cooperation with countries where Akabane virus is endemic in order to compare and share virological, epidemiological and entomological information.

## Conclusion

A large-scale outbreak of the disease characterized by neurological symptoms such as astasia, ataxia, tremor, nystagmus, opisthotonus and hypersensitivity occurred in beef and dairy cattle in southern Japan from summer to autumn in 2006. Nonsuppurative encephalomyelitis was observed in these clinical cases by histopathological findings. Akabane viral antigen and genome were detected in the central nervous system of the affected cattle. Akabane virus was isolated not only from them but also from the blood samples of cohabitated and sentinel cattle in the epidemic area. The isolates were closely related to the Iriki strain in genogroup I a, which caused encephalitis in calves in 1984. Most of the affected cattle possessed the neutralizing antibody against Akabane virus. Seroconversion of the sentinel calves was also confirmed during an outbreak of the disease. No evidence of Akabane virus circulation was observed in 2005 in the nation-wide serological surveillance, suggesting that a new strain belonging to genogroup I a invaded southern Japan from overseas in the summer of 2006 and caused an epizootic of encephalomyelitis in cattle. It is recommended that an advanced vaccine be developed to control the disease properly.

## Methods

### Processing of tissues for histopathology and immunohistochemistry

Tissue samples of central nervous systems were collected from 72 affected cattle. These were fixed with 10% buffered formalin and processed for paraffin-wax embedding. The tissue sections from each paraffin block were stained with hematoxylin and eosin for histopathological examination. One pair of serial sections from cattle that showed neurological symptoms was submitted for immunohistochemical assessment of the detection of Akabane viral antigens. After deparaffinization of the pair sections, endogenous peroxidase activity was inhibited by treatment with 3% H_2_O_2 _in absolute methanol for 20 min at room temperature. In each section pair, one section was incubated with a rabbit antiserum (1:2000 dilution) against the Akabane virus OBE-1 strain [31], while the other was not incubated with the antiserum as a negative control. The sections were then labeled using a HISTOFINE SAB-PO kit (Nichirai Co., Tokyo) containing a biotinylated secondary antibody for anti-rabbit IgG and streptavidin-conjugated peroxidase. Localized peroxidase conjugates were visualized with 3, 3'-diaminobenzidine. All sections were lightly counterstained with hematoxylin.

### Extraction and amplification of viral genome from clinical samples

Tissue specimens obtained from the brain and spinal cord of affected cattle were rinsed, minced and homogenized in serum-free Eagle's minimum essential medium (MEM) (Nissui Pharmaceutical Co., Tokyo, Japan). The supernatant of a 10% homogenate of each tissue was used for viral RNA preparation. Total RNA was extracted using the QIAamp Viral RNA Mini kit (Qiagen, Valencia, CA, USA). The primer pairs AKAI172F/AKAI560R (5'-CAGAAGAAGGCCAAGATGGT-3'/5'-AAGTTGACATCCATTCCATC-3') and the QIAGEN OneStep RT-PCR kit (Qiagen) were used for detecting the viral S RNA segment. Reverse transcription was conducted at 50°C for 30 min. This mixture was then heated for 95°C for 15 min to stop the reaction and to activate HotStart *Taq *DNA polymerase. The resulting cDNA was amplified by 35 cycles of denaturation at 94°C for 30 s, annealing at 55°C for 30 s and extension at 72°C for 45 s followed by one step of final extension at 72°C for 10 min. The PCR products were electrophoresed on a 1.5% agarose gel and visualized by staining with ethidium bromide.

### Virus isolation

The supernatant described above was also used for virus isolation. On the other hand, blood samples were collected from clinically healthy cattle in the affected farms and from sentinel calves. Heparinized blood samples were separated into erythrocytes and plasma by centrifugation, and the erythrocytes were washed three times with cold PBS. The supernatant of 10% homogenates of tissues, plasma and washed erythrocytes were stored at -80°C until use. Several blood samples were directly used as inoculum after being frozen and thawed once. These specimens were inoculated into monolayer cultures of hamster lung (HmLu-1) and/or baby hamster kidney (BHK-21) cells. After incubation at 37°C for 7 days, the cell culture fluids were pooled and subinoculated into freshly prepared cell cultures at least twice until the infected cells exhibited the cytopathic effect.

### Immunobinding assay

Monoclonal antibodies recognizing the N protein and seven neutralizing epitopes (A1, A2, B, C1, C2, D and E) on the Gc protein of Akabane virus strain OBE-1 were used for antigenic characterization of the isolates [25]. The supernatants of virus-infected cells were blotted onto an Immobilon-P membrane (MILLPORE, Bedford, MA, USA) using the blotting apparatus with 9 cm-long slots. The membrane was immersed in blocking buffer (8% skimmed milk powder in TBS; 20 mM Tris-HCl pH 7.5, 150 mM NaCl) at 4°C overnight. Then the membrane was turned 90 degrees around and placed again into the same blotting apparatus described above. Each diluted monoclonal antibody was added to a slot and incubated for 1 h at room temperature on an orbital shaker. After three washes in TBS, the membrane was reacted with horseradish peroxidase conjugated goat antibody to mouse IgG (Cappel/ICN, Aurora, OH, USA) for 30 min at room temperature and rinsed five times with TBS. The immune complexes were detected by color development with 3, 3'-diaminobenzidine tetrahydrochloride as a substrate.

### Virus neutralization test

Bovine sera were serially diluted twofold with serum-free MEM in the 96-well cell culture microplates. Each serum dilution was mixed with an equal volume of 200 50% tissue culture infective doses/0.05 ml of virus and incubated at 37°C for 1 h. Then 0.1 ml volumes of HmLu-1 cells suspended in GIT medium (Wako Pure Chemical Industries, Ltd., Osaka, Japan) were added to each well. After incubation at 37°C for 7 days, the antibody titer was determined as a reciprocal of the highest serum dilution showing complete inhibition of the cytopathic effect. For a cross-neutralization test, rabbit immune sera to the OBE-1, Iriki and KM-1/Br/06 strains of Akabane virus were produced by the method described previously [22] with the approval of the Animal Ethics Committee, National Institute of Animal Health (11 June, 2007), under approval number 07-60.

### RT-PCR, sequencing and phylogenetic analysis

Viral RNA was extracted from the supernatant of virus-infected cells using the High Pure Viral RNA kit (Roche Diagnostics, Mannheim, Germany) for sequencing. Oligonucleotide primers AINO-5' (5'-AGTAGTGTGGCTCCAC-3') and AINO-3' (5'-AGTAGTGTACTCCACTAT-3') were used for RT-PCR of the S RNA segment. These primers corresponding to the terminal sequences of the Aino virus S RNA segment are available for specific amplification of the corresponding gene of some other species including Akabane virus [26,32]. The full-length M RNA segment was amplified by RT-PCR using the primer AKAMEND21 (5'-AGTAGTGWWCTACCACAACAA-3') [27]. The cDNAs of S and M RNA segments were synthesized and amplified with the Titan One Tube RT-PCR kit (Roche Diagnostics) under the conditions described previously [26,27]. The PCR fragments were purified by the QIAquick PCR purification kit (Qiagen) and directly sequenced in both orientations with sequencing primers specific to the S and M RNA segments [26,27], respectively, of Akabane virus using the BigDye Terminator v3.1 sequencing kit (Applied Biosystems, Foster City, CA, USA) and the ABI3100-Avanti Genetic Analyzer (Applied Biosystems). Nucleotide and amino acid sequences were analyzed by DNASIS Pro version 2.0 (Hitachi Software Engineering, Co. Ltd., Tokyo, Japan) and the University of Wisconsin Genetic Computer Group (UWGCG) package. The sequences were aligned by the CLUSTAL W program [33]. The phylogenetic trees of the coding regions in the S and M RNA segments were constructed by the neighbor-joining method [34] using Kimura's two-parameter formula, and the reliability of the branching orders was evaluated by the bootstrap test (n = 1000). The resulting dendrogram was viewed and edited using the Tree View program [35].

### Nucleotide sequence accession numbers

The nucleotide sequence data reported in this article were deposited in the DNA Data Bank of Japan (DDBJ) with the accession numbers AB373232, AB373234, AB426271–AB426280 (for the S RNA segment) and AB373233, AB426281–AB426282, AB436954 (for the M RNA segment). The following previously published nucleotide sequence data were used in this study: AB000819, AB000851–AB000854, AB000857, AB000863, AB000864, AB000867, AB232182, AB232191–AB232193, AB232196–AB232198, AB232200, AB232204, AB232207, AB232210, AB232213, AB232214, AB232218, AB232222, AB232231, AB232240, AB232243, AB232251, AB232260, AB232264, AB232265, AB232268, AB232299, AB232314, AB232319, AB232320, M22011 for analysis of the S RNA segment and AB100604, AB100605, AB208700, AB297818–AB297851 for analysis of the M RNA segment.

## Authors' contributions

RK, MH and SI detected viral genome and isolated viruses from clinical samples of bovine encephalomyelitis, and performed the serological survey. MK, YG and ST carried out histopathological and immunohistochemical investigations. TY contributed to the comparative sequence analysis and the construction of phylogenetic trees of the isolates. TK contributed to the production of rabbit antisera and the antigenic characterization of the isolates. TT participated in the epidemiological analysis of the disease. TI coordinated the experiments and helped to draft the manuscript. MY designed the experiments, analyzed the data and drafted the manuscript. All authors have read and approved the final manuscript.
